# Wealth-based inequality in maternal health service utilization among ever-married women of reproductive age in Somaliland; secondary analysis of the 2020 Demographic and Health Survey

**DOI:** 10.3389/fpubh.2026.1899022

**Published:** 2026-08-19

**Authors:** Abdirahman A. Suleiman, Aline Semaan

**Affiliations:** 1 Department of Public Health, Adal University, Hargeisa, Somaliland, Somalia; 2 Department of Policy, Planning and Strategic Information, Ministry of Health, Hargeisa, Somaliland, Somalia; 3 Sexual and Reproductive Health Unit, Institute of Tropical Medicine Antwerp, Antwerp, Belgium

**Keywords:** concentration index, cross-sectional, Demographic and Health Survey, maternal health services, slope index of inequality, Somaliland, sub-Saharan Africa, wealth-based inequality

## Abstract

**Background:**

Access to affordable, quality maternal health services is a fundamental human right. In Somaliland, characterized by a fragile health system and high maternal mortality, utilization of maternal health services remains low, and socioeconomic factors are known to shape this utilization. However, the extent and implications of wealth-based inequalities in maternal service use remain poorly quantified. This study assesses the magnitude of wealth-based inequality in adequate antenatal care (ANC) and public facility-based childbirth in Somaliland.

**Methods:**

This secondary analysis used the 2020 Somaliland Demographic and Health Survey, including 3,065 ever-married women with a recent birth. Adequate ANC was defined as four or more visits, consistent with Somaliland's Focused ANC Model at the time of the survey rather than the current WHO-recommended eight contacts. Private facility births were excluded, as they are concentrated among wealthier, urban women and fall outside Somaliland's free public maternal care policy; we therefore assessed births in public facilities, which provide free care to the general population. We used survey-weighted logistic regression incorporating sampling weights, clustering, and stratification, and quantified inequality using the Slope Index of Inequality (absolute) and Wagstaff's concentration indices corrected for binary outcomes (relative).

**Results:**

The weighted prevalence of adequate ANC was 20%, and 32% of women gave birth in a public facility. After adjustment, the richest women had nearly four times higher odds of adequate ANC (aOR: 3.98; 95%CI: 2.20–7.17) and over six times higher odds of public facility-based birth (aOR: 6.54; 95%CI: 3.59–11.91) than the poorest. Adjusted absolute differences between the richest and poorest quintiles were 26 percentage points for ANC and 49 percentage points for public facility births. Significant pro-rich distribution was observed for both outcomes, with concentration indices of 0.34 (95%CI: 0.33–0.35) for ANC and 0.50 (95%CI: 0.49–0.52) for public facility births.

**Conclusion:**

Wealth-based inequalities exist in maternal health service utilization in Somaliland. Addressing these inequalities requires targeting poorer, non-urban women and removing access barriers through systematic reforms beyond the health system

## Introduction

According to recent UN estimates, the maternal mortality ratio in Sub Saharan Africa (SSA) has declined from 748/100,000 livebirths in 2000 to 454/100,000 livebirths in 2023 (1). Antenatal care (ANC) and facility-based births are two key maternal health interventions that have likely contributed to this reduction (2–6). A systematic review estimated that ensuring skilled attendance at every birth could avert between 16% and 33% of maternal deaths, primarily through the timely detection and management of intrapartum complications (7); antenatal care, in turn, provides a critical platform for early risk detection, referral, and birth preparedness that complements this effect (8). However, the coverage of 4 or more ANC visits and facility-based births in SSA has been reported as 57% and 64% respectively, according to 2023 estimates from UNICEF, and both of them are below the global level coverages (9, 10).

Additionally, substantial wealth-based inequalities in utilization of maternal health services persist in SSA. A growing body of evidence from countries including Benin, Burkina Faso, Ethiopia, Guinea, Malawi, Niger, Senegal, Somalia, Togo, Zambia, and Zimbabwe reported wealth-based inequality in the utilization of ANC visits (11–23). Similarly, studies from Botswana, Democratic Republic of Congo, Ethiopia, Ghana, Mozambique, Namibia, Somalia, and Tanzania indicated patterns of pro-rich inequality in facility-based childbirth care utilization where women from richer population-segments have higher odds to give birth in a health facility compared to the poorer population (3, 4, 16, 20, 23–30). This disparity in access continues to exist despite the efforts of many governments to strengthen primary healthcare and promote universal access to essential maternal and child health services as outlined in the SDGs (31–33).

The literature consistently indicates that wealth-based inequality – which is unfair, unjust and avoidable– is one of the strongest drivers of unequal access to essential maternal health services in SSA; and is impairing the progress of the Universal Health Coverage (UHC) (34–37). Additionally, the evidence suggests that eliminating within-country wealth-based inequalities could increase coverage of reproductive, maternal, newborn, and child health interventions by 10 to 20 percentage points, pointing out the need for policies that deliberately prioritize the most disadvantaged populations (35).

In Somaliland, maternal service utilization remains low, with only 20% of women reporting four or more ANC visits and 33% giving birth in a health facility, according to the 2020 Somaliland Demographic and Health Survey (38). The low maternal health utilization rates points–and at the same time the result of–to a health system that faces distinct structural constraints relative to many other SSA settings, including a limited health workforce, dispersed rural and nomadic populations, recurring drought and clan-based insecurity in parts of the country, all of which shape access to maternal health services (39–42). Some studies–from the local context–have modeled the association between socioeconomic factors, including household wealth, and maternal health service utilization in Somaliland (23, 39, 42, 43), demonstrating that household wealth is associated with higher odds of ANC utilization and giving birth in a facility. Regression-based determinant analysis identifies statistical associations between covariates and outcomes, but it does not quantify the size of the wealth-related gap in coverage, nor does it yield a single, standardized metric that can be compared across studies, settings, or time.

However, none of these studies have applied inequality metrics to estimate the magnitude of wealth-based inequality in maternal health service utilization. As a result, the extent of inequality in maternal health service use remains insufficiently quantified in Somaliland. Starting from this gap, absolute and relative measures of inequality address this gap by explicitly ranking the population along the wealth distribution and quantifying how service coverage is distributed across that gradient; and this study will use these measures.

The objective of this study is to, firstly, assess the coverage of adequate antenatal care and public facility-based births. Secondly, it aims to examine the socioeconomic determinants of these two maternal health indicators. And finally, it quantifies wealth-based inequality in adequate ANC visits and public facility-based births using both absolute and relative measures of inequality.

## Methods

### Study design and data

This is a cross-sectional quantitative study using secondary data from the Somaliland 2020 Demographic and Health Survey (SLDHS 2020), which is a nationally representative household survey. The SL-DHS2020 included a sample of 6,285 women of reproductive age (38). The SL-DHS 2020 used a multistage stratified cluster sampling design. A sampling frame of 2,806 primary sampling units (PSUs) was developed for urban and rural areas. In the first stage, 483 PSUs were selected using probability proportional to size: 208 urban, 190 rural, and 85 nomadic. In the second stage, secondary sampling units were drawn using the same method, and 30 households were systematically selected from each PSU. For nomadic communities, temporary settlements were identified with the help of clan elders and nomadic link workers. These settlements served as PSUs and followed a two-stage sampling process. In smaller nomadic sites, all eligible households were included to avoid underrepresentation (38).

### Study population and sample

This study used data from the 2020 Somaliland Demographic and Health Survey (SLDHS), a nationally representative household survey designed to produce estimates of key health indicators across urban, rural, and nomadic populations. The SLDHS employed a multistage stratified cluster sampling design, with stratification by residence and by the six administrative regions of Somaliland (Awdal, Marodijeh, Sahil, Togdheer, Sool, and Sanaag). The survey targeted women aged 15–49 years residing in sampled households, using probability proportional to size approach maintaining representativeness across population strata.

For this analysis, the study population was restricted to ever-married women of reproductive age, as only this group was asked detailed questions on pregnancy and childbirth in the survey. To capture recent maternal health service utilization and minimize recall bias, the sample was further limited to women whose most recent live birth occurred within the five years preceding the survey. Although information on place of delivery was collected for all birth; however, in this study, both indicators for antenatal care and place of delivery analyses were consistently restricted to the most recent birth.

**Figure 1:**
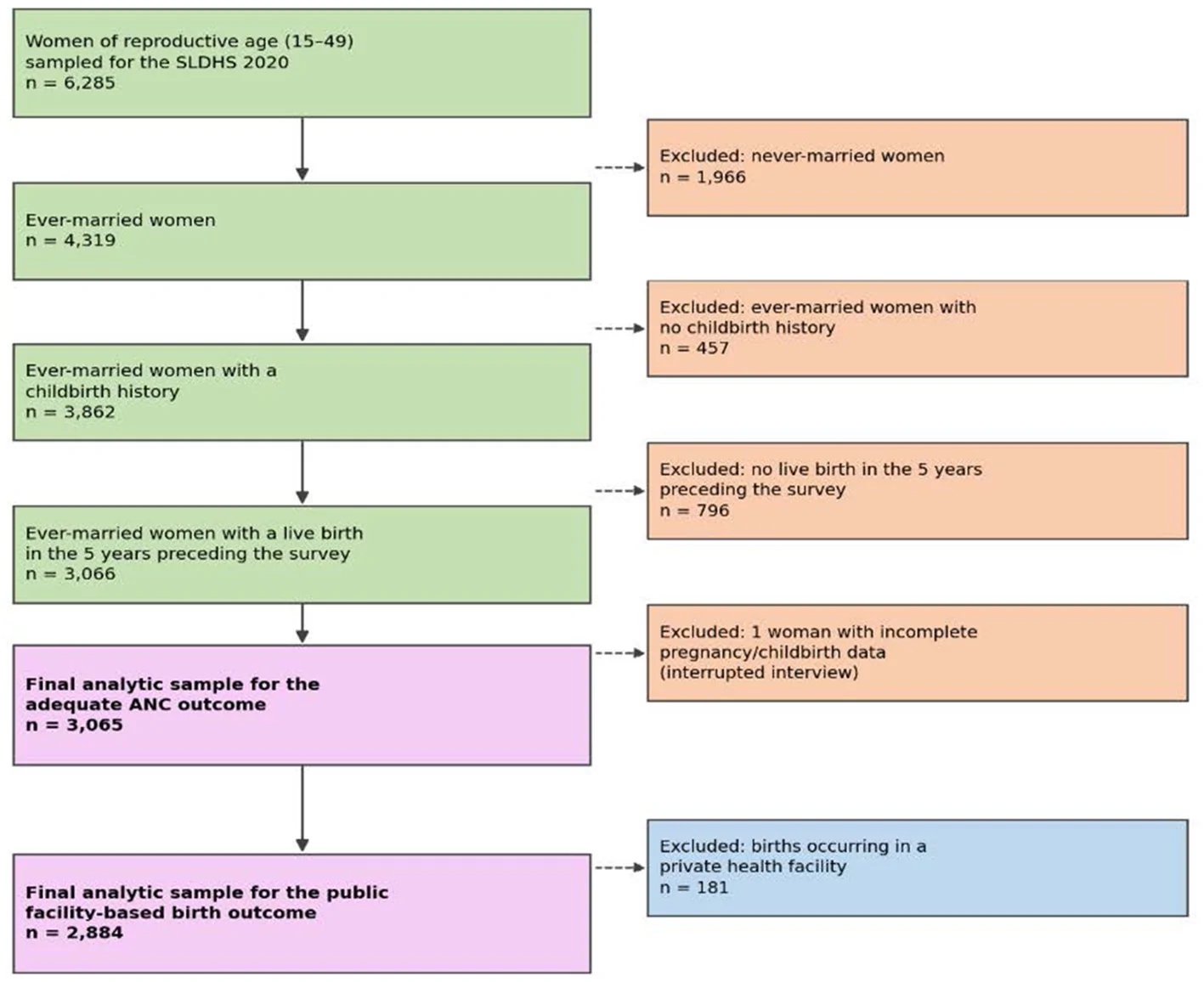
Study population diagram. Derivation of the analytic sample from the 2020 Somaliland Demographic and Health Survey.

Of the 6,285 women interviewed in the SLDHS, 3,065 ever-married women with a livebirth in the five years preceding the survey were included in the final analytic sample. For the analysis of public facility-based childbirth, women who delivered in private facilities were excluded, in line with the study's focus on access to public maternal health services, resulting in a final denominator of 2,884 women for this outcome. All analyses accounted for the complex survey design and sampling weights to produce estimates representative of the population that account for the DHS' sampling strategy. See Figure 1 below.

### Variable definition

#### Outcome variables

Two essential maternal health services during pregnancy and childbirth were considered outcome variables for this analysis: *(i) Adequate ANC;* defined as 4 or more ANC visits during the most recent pregnancy. While the WHO currently recommends a minimum of eight ANC contacts, Somaliland's national antenatal care protocol at the time of the survey followed the earlier “Focused ANC Model,” which recommends at least four visits; we adopted the four-visit threshold as the applicable national standard of adequate care during the study period. Those respondents who replied “don't know” in the number of ANC visit made in their last pregnancy were considered as having one ANC visit based on the assumption that women are less likely to forget if they had at least one ANC visit, i.e., one ANC visit is in the category of “inadequate ANC visit” (13).

*(ii) Public facility-based births;* we intentionally focused on all births that occurred in a public health facility for two reasons: *(a)* public facilities in Somaliland offer free maternal healthcare services, based on the free maternal care policy designed to improve equitable access. Therefore, we aimed to assess if this policy is achieving its equity-oriented objectives. *(b)* Private provision is cost-based and largely utilized by wealthier women in urban setting and, therefore, we excluded the women who sought childbirth care in the private sector from the reference group. In other words, Because private maternity care is fee-based, women who deliver privately are plausibly concentrated among the wealthiest households; excluding them therefore has two implications: it strengthens our ability to assess equity specifically within the public, nominally free system, but it also means our public-facility outcome under-represents institutional delivery coverage overall, since it does not capture the (largely wealthier) births occurring privately. We do not have facility-type-stratified wealth data in the present dataset to quantify this directly, and we return to this point as a limitation in the Discussion. This outcome variable was, finally, coded 1 for births that took place in a public health facility and 0 for births that took place outside a public health facility.

#### Equity identifier

*Household wealth index*: The wealth index is a composite score calculated using principal component analysis based on a household's ownership of selected assets including televisions, bicycles and cars; materials used for housing construction; types of water access; sanitation facilities and types of fuel used. This continuous scale of relative wealth was then categorized into quintiles: lowest (quintile 1, labeled, poorest), second (quintile 2, poorer), middle (quintile 3, middle), fourth (quintile 4, richer) and highest (quintile 5, richest). This variable is precalculated and available in the dataset and we used it as it is.

#### Independent variables

Based on the WHO's social determinants of health conceptual framework–which was used to explain inequality–and published articles, we have selected explanatory variables for our analysis. The framework explains structural and intermediary determinants impacting equity in health. Based on these determinants, we identified several factors that influence utilization of services and shape equality in health and they are as follows:

1. **Wealth Index:** The primary exposure variable in this study is the household Wealth Index, which reflects the relative living standards of women's households. It is computed by the DHS program based on the ownership of selected assets using Principal Component Analysis (PCA) and is categorized into quintiles: poorest, poorer, medium, richer, and richest.
2. **Type of Residence:** The type of residence of the participant was categorized into three groups by the DHS: urban, rural, and nomadic. It is worth noting that the distinction between rural and nomadic populations is that nomadic communities are constantly moving in search of better grazing for their livestock, unlike rural populations, which are typically settled in one location.
3. **Region of Residence:** This variable identifies the administrative region of Somaliland where the respondent resides at the time of the interview. Somaliland is divided into six regions: Awdal, Marodijeh, Sahil, Togdher, Sool, and Sanaag.
4. **Mother's Education:** The highest level of education attained by the respondent was categorized into four groups: (a) no education, (b) primary education, (c) secondary education, and (d) tertiary education. However, for the purposes of this study, this variable was dichotomized the variable into “not educated” and “educated.” This decision was based on two factors: (a) the small sample sizes in the educated categories, and (b) the assumption that any form of education–whether primary or above–is likely to affect the individual's cognitive and perception toward healthcare seeking (44–47).
5. **Partner's Education:** Similar to the respondent's education, this variable indicates the highest level of education attained by the partner. Like the mother's education variable, this variable was also dichotomized into “not educated” and “educated” for consistency with the previous reasoning.
6. **Parity Prior to the Last Pregnancy:** Parity is the number of birth events the mother has experienced which may or may not be equal to the total number of children. Multiple births, such as twins or triplets, were counted as a single birth event. Parity was calculated using variables of birth records and multiple birth records from the DHS dataset, followed by subtracting 1 from the new variable to capture parity prior to the most recent childbirth. Parity was then categorized into three groups: 0, 1–3, and 4+, reflecting the conventional distinction in the maternal health literature between nulliparous, low-parity, and high-parity (grand multiparous) women, groups that are known to have differing obstetric risk profiles and care-seeking patterns.
7. **Decision-Maker for Seeking Care:** This variable (V743A in the DHS dataset) originally categorized responses into (a) respondent, (b) husband, (c) jointly, (d) siblings, and (e) others. For the analysis, it was re-categorized into two groups: (a) “Either by the Respondent Independently or Jointly with the husband” and (b) “Husband and others only” (where the mother was not part of the decision-making). It was assumed that decision-making of the mother is a contributory factor of empowerment and independency (30, 48).
8. **Age of the mother at the time of the most recent livebirth:** This variable captures the mother's age at the time of her last pregnancy, calculated by subtracting the mother's current age (V012) from the birth date of her last child (B301). The resulting age was then categorized into three groups: 15–19 years, 20–29 years, and 30 years and above, consistent with standard maternal-age risk categories used in the DHS-based maternal health literature to distinguish adolescent mothers from women in the primary and later reproductive years.

### Data analysis

All analyses were conducted with the appropriate sampling and design weights, which combined individual women's weights with cluster and strata adjustments. Descriptive statistics were performed, presenting unweighted counts, weighted percentages, and the 95% confidence intervals. We also presented, through cross-tabulation, the distribution of outcome variables according to exposure variables, reporting both weighted percentages and unweighted counts.

Subsequently, we did bi-variate analysis between each outcome variable and the exposure variables, and we reported odds ratios, 95% confidence intervals (CI), and *p*-values based on Wald tests. For the multivariable analysis, we initially included all variables with a Wald test *p*-value equal to or less than 0.1 into one model and then reported the results. In the second model, we retained only those variables with a significant Wald test *p*-value less than 0.05 we used this two-step, theory-informed selection approach, retaining covariates identified a priori from the WHO social determinants of health framework while allowing data-driven refinement, rather than a fully automated stepwise procedure. Adjusted Wald tests were used to determine whether excluding a variable significantly changed the model's fitness of data until we reached the final model. After finalizing each of the two outcomes' regression models, we tested interaction effects between the remaining explanatory variables using the Wald test.

The final step involved calculating the extent of wealth-based inequality using the Slope Index of Inequality (SII) and concentration indices and curves. The SII was calculated first by regressing each binary outcome on a Ridit-scaled wealth rank using survey-weighted generalized linear models with an identity link; it represents the absolute difference in predicted coverage between the poorest (rank = 0) and richest (rank = 1). A positive SII indicates higher utilization among wealthier women and a negative SII indicates a pro-poor distribution (49). This was followed by plotting concentration curves. Concentration curves were constructed by ranking women by their DHS wealth index and plotting the cumulative share of the population (x-axis) against the cumulative share of the health outcome using survey weights (y-axis). The degree of inequality is visually assessed by comparing the curve with the line of equality.

Wagstaff's concentration indices were then estimated to quantify the relative degree of wealth-based inequality. A positive concentration index indicates that the outcome is concentrated among wealthier individuals, while a negative index indicates concentration among poorer individuals (50, 51). The adjusted concentration indices (derived from final regression models incorporating survey weights and covariates) were reported.

The formula used to calculate Wagstaff's concentration index were:


$$\begin{array}{l} CI=\frac{2}{\mu }\;.\;cov\left(y,r\right) \end{array}$$ (1)

where *y* is the health outcome, *r* is the fractional rank of individuals in the wealth distribution, and μ is the mean of the outcome. The resulting index takes values between −1 and +1. We applied Wagstaff's correction of the concentration index, which is appropriate for binary outcomes (50). Data analysis was conducted using RStudio version 4.5.1.

### Missing data

Women with missing pregnancy or childbirth information were excluded from the analysis. This included women who had never married, had never given birth, or whose most recent live birth occurred more than five years before the survey, as well as one respondent whose interview appeared incomplete; these exclusions are shown in the population in Figure 1. Two more variables—partner's education and decision-making for seeking care—had missing values. For partner's education, 277 women had missing data; 205 were divorced or widowed and therefore skipped the question, while 72 provided no response for unclear reasons. For decision-making on seeking care, 32 women did not answer the question. These missing cases were reported in the descriptive analysis and excluded from the bivariate and multivariable regression models. Missingness in these two variables was concentrated among divorced or widowed women (for partner's education) and among a small number of non-responses (for decision-making); their exclusion from the regression models may have modestly reduced statistical power for these specific covariates and could introduce small biases if marital status or care-seeking circumstances were also linked to the outcomes. However, given that missingness affected under 10% of observations for partner's education and about 1% for decision-making, we do not expect this to have materially altered the main wealth-based inequality findings, which are the primary focus of this analysis.

### Ethics

This study received ethical approval from the Institutional Review Board (IRB) of the Institute of Tropical Medicine, Antwerp, Belgium (Reference No. 1865/25). Authorization to use the Somaliland Demographic and Health Survey (DHS) dataset for secondary analysis was obtained from the Government of Somaliland.

The study utilized anonymized secondary data; therefore, additional informed consent from participants was not required. At the time of the original survey, written informed consent was obtained from all participants prior to data collection, as documented in the DHS report. The original DHS protocol also received ethical approval from the relevant national ethics committee.

## Results

### Sociodemographic characteristics of study population

The study population comprises 3,065 ever-married women aged 15–49 with a median age at the childbirth of 20 years. The inclusion and exclusion criteria for the study population is shown in Figure 1 above. Table 1 describes the distribution of the sample by sociodemographic characteristics. In terms of socioeconomic status, 25% belonged to the poorest wealth quintile, while 31.7% were from the richest quintile. Most women had no formal education (77.1%) and had given birth to four or more children prior to their last pregnancy (44%). In terms of decision-making on care-seeking, 66.1% of women reported either they decided independently or jointly with their husbands. The largest percentage of the sample were from Marodijeh (29.3%) and the smallest were from Sahil (5.5%) (Table 1).

**Table 1:** Sociodemographic characteristics of ever-married women who had a most recent livebirth during the past 5 years preceding the SL-DHS2020 in Somaliland.

Sociodemographic variables	Number (*n* = 3,065)	Weighted % (95% CI)
Wealth index		
Poorest	1,116	25.0% (21.0%−29.6%)
Poorer	506	13.3% (9.9%−17.6%)
Middle	340	11.5% (9.2%−14.4%)
Richer	498	19.4% (16.2%−23.2%)
Richest	605	31.7% (25.3%−36.7%)
Residence type		
Urban	864	48.8% (42.6%−55.0%)
Rural	1,037	26.5% (19.9%−34.4%)
Nomadic	1,164	24.7% (21.5%−28.2%)
Region of residence		
Awdal	387	9.0% (6.4%−12.7%)
Marodijeh	377	29.3% (23.1%−36.4%)
Sahil	387	5.5% (3.9%−7.5%)
Togdheer	514	25.7% (20.5%−31.7%)
Sool	681	13.2% (9.7%−17.7%)
Sanaag	719	17.3% (14.4%−20.1%)
Mother's education		
No formal education	2,520	77.1% (74.2%−79.7%)
At least has a primary education	545	22.9% (20.3%−25.8%)
Partners' education		
No formal education	1,975	62.0% (58.3%−65.8%)
At least has a primary education	813	37.9% (34.2%−41.7%)
Missing Data (NA)	277	
Parity Prior to the Last Pregnancy		
0	378	13.1% (11.2%−15.3%)
1–3	1,334	42.9% (40.2%−45.5%)
4 and more	1,353	44.0% (41.3%−46.8%)
Who decides to seek care, in general		
Independently by the respondent or in consultation with her husband	1,864	66.1% (64.0%−68.1%)
Husband and/or Others without mother's engagement	1,169	33.9% (31.9%−36.0%)
Missing Data (NA)	32	
Age of the mother at the time of the most recent livebirth (in groups)		
15–19	1,309	40.2% (37.7%−42.7%)
20–29	1,643	55.3% (53%−57.6%)
30 and above	113	4.5% (3.6%−5.6%)

### Coverage of maternal health service

Our analysis showed that 1 in 5 women [20% (95%CI 17%−23%)] made 4 or more ANC visits during their last pregnancy, i.e., adequate ANC visits. For the place of birth, our analysis showed that the percentage of public health facility-based childbirths were 32% (95%CI: 24%−36%).

### Coverage of adequate ANC visits and public health facility childbirths by socioeconomic characteristics: analysis of the 2020 Somaliland DHS

A clear wealth quintiles gradient in both adequate ANC visits and public health facility-based births is observed where women in the richest quintile had the highest levels of adequate ANC utilization (34%) and public health facility-based childbirths (60.6%), compared to only 3.5% and 6.6%, respectively, among the poorest quintile.

Adequate ANC coverage was higher in urban areas (30.4%), among women with at least a primary education (32.9%), and those with no prior births (22.9%) and those who participated in care-seeking decisions (22.5%) and in Awdal, Marodijeh, and Togdheer (28.5%, 26.7% and 25.6%, respectively). Public facility childbirths was similarly highest in urban areas (53.8%), compared with rural (25.9%) and nomadic populations (4.8%). Awdal (46.9%) and Marodijeh (51.3%) had the highest utilization of public facility-based childbirths, whereas Sool (13.9%) and Sanaag (12%) remained the lowest. Public facility childbirths was more common among women with at least a primary education (55.9%), those with educated partners (52.8%), women involved in decision-making (36.4%), and primiparous women (43.5%) (Table 2).

**Table 2:** Distribution of adequate ANC visits and public health facility-based births utilization by background characteristics among ever-married women who had a most recent livebirth during the past 5 years preceding the SL-DHS2020 in Somaliland.

Sociodemographic determinants	1–Adequate ANC Visits (*n* = 3,065)		2-Public health facility-based births (*n* = 2,884)	
	Adequate ANC visits (4+ ANC)	Weighted (95% CI)	Gave birth in a public health facility	Weighted % (95% CI)
Wealth index				
Poorest	34	3.5% (2.2%−5.6%)	77	6.6% (5.2%−8.5%)
Poorer	45	10.2% (6.1%−16.7%)	65	14.1% (10.0%−19.3%)
Middle	55	22.9% (15.7%−32.1%)	94	34.9% (27.9%−42.6%)
Richer	105	24.1% (19.4%−29.7%)	180	45.0% (37.1%−53.2%)
Richest	186	34.0% (28.9%−39.7%)	264	60.6% (53.4%−67.4%)
Residence type				
Urban	230	30.4% (25.9%−35.2%)	364	53.8% (48.8%−58.8%)
Rural	182	18.7% (12.5%−26.9%)	258	25.9% (17.8%−36.2%)
Nomadic	13	1.0% (0.5%−1.9%)	58	4.8% (3.6%−6.2%)
Region				
Awdal	98	28.5% (23.7%−33.8%)	147	46.9% (36.1%−57.9%)
Marodijeh	68	26.7% (19.3%−35.3%)	102	51.3% (36.3%−66.1%)
Sahil	72	18.4% (14.4%−23.1%)	160	45.2% (37.6%−53.0%)
Togdher	90	25.6% (18.3%−35.6%)	95	29.5% (22.0%−38.3%)
Sool	43	6.3% (4.7%−8.6%)	92	13.9% (11.0%−17.5%)
Sanaag	54	7.2% (5.4%−9.7%)	84	12.0% (8.7%−16.5%)
Education level of the respondent				
No formal Education	277	16.2% (13.3%−19.6%)	463	26.1% (22.2%−30.3%)
At Least has a Primary Education	148	32.9% (26.9%−39.5%)	217	55.9% (49.1%−62.6%)
Husband's level of education				
No formal education	175	13.8% (11.4%−16.6%)	302	21.7% (18.3%−25.5%)
At Least has a Primary Education	206	29.1% (24.9%−33.8%)	314	52.8% (45.8%−59.7%)
Women's Parity prior to the last pregnancy				
0	64	22.9% (16.5%−30.8%)	118	43.5% (34.5%−52.9%)
1 – 3	195	22.2% (17.9%−27.1%)	313	33.5% (28.1%−39.6%)
4 and more	166	17.1% (14.0%−20.6%)	249	26.8% (22.4%−31.7%)
Who decides to seek care, in general				
Independently by the respondent or in consultation with her husband	309	22.5% (19.1%−26.3%)	464	36.4% (31.4%−42.6%)
Husband and other, excluding mother	110	14.8% (11.3%−19.2%)	211	23.4% (19.2%−28.1%)
Age of the mother at the time of the most recent live birth (in groups)				
15 – 19	150	19.0% (14.9%−23.9%)	237	26.5% (21.2%−32.5%)
20 – 29	255	21.0% (17.1%−25.6%)	414	35.6% (31.3%−40.2%)
30 and above	20	17.2% (8.5%−31.9%)	29	32.9% (21.2%−47.1%)

### Multivariable regression analysis

Table 3 presents the adjusted multivariable regression results for adequate ANC visits and public facility-based childbirth. After adjustment, women in the richest households had nearly four times higher odds of receiving adequate ANC [aOR: 3.98 (95% CI: 2.20–7.17)] compared to the poorest, while nomadic women had notably lower odds [aOR = 0.10 (95% CI: 0.04–0.21)] compared to urban women. In terms of Region-wise, women from Awdal region showed the highest adjusted odds of utilizing adequate ANC visits [aOR = 1.81 (95% CI: 1.14–2.88)], whereas women from Sool and Sanaag had substantially lower odds [aOR = 0.42 (95% CI: 0.23–0.75] and aOR = 0.40 [95% CI: 0.24–0.67)], respectively. Other factors including maternal and partner education, parity, decision-making autonomy, and age, were not significant in the ANC model and were excluded from the final specification.

**Table 3:** Multivariable logistic regression analysis of adequate ANC Visits and public facility-based births among ever-married women who had a most recent livebirth in the 5 years preceding the SL-DHS2020 in Somaliland.

Multivariable logistic regression results				
Sociodemographic determinants	Adequate ANC Visits (4+ ANC Visits) (*n* = 3,065)		Public Health Facility-based Childbirths (*n* = 2,884)	
	aOR (95% CI) regression model (final model)	*P* value	aOR (95% CI) regression model (final model)	*P* value
Wealth index				
Poorest	Ref	–	Ref	-
Poorer	1.57 (0.81–3.05)	0.179	1.79 (0.99–3.25)	0.054
Middle	2.60 (1.34–3.05)	0.004	3.32 (1.94–5.66)	< 0.001
Richer	2.82 (1.49–5.31)	0.001	4.87 (2.47–9.55)	< 0.001
Richest	3.98 (2.20–7.17)	< 0.001	6.54 (3.59–11.91)	< 0.001
Residence type				
Urban	Ref	–	Ref	-
Rural	0.87 (0.57–1.33)	0.530	0.43 (0.28–0.65)	< 0.001
Nomadic	0.10 (0.04–0.21)	< 0.001	0.21 (0.13–0.34)	< 0.001
Region of residence				
Marodijeh	Ref	–	Ref	-
Awdal	1.81 (1.14–2.88)	0.013	1.42 (0.79–2.56)	0.234
Sahil	0.93 (0.58–1.47)	0.750	1.17 (0.66–2.08)	0.577
Togdheer	1.18 (0.72–1.93)	0.516	0.32 (0.18–0.56)	< 0.001
Sool	0.42 (0.23–0.75)	0.004	0.27 (0.15–0.46)	< 0.001
Sanaag	0.40 (0.24–0.67)	< 0.001	0.16 (0.08–0.30)	< 0.001
Parity prior to the last pregnancy				
0 parity	–		Ref	–
1–3 parities	–		0.65 (0.42–1.00)	0.048
4+ parities	–		0.48 (0.29–0.76)	0.002
Education Level of the mother				
No formal education	–		Ref	–
At Least has a Primary Education	–		1.78 (1.23–2.57)	0.002

For public facility births, women in the richest quintile were more than six times higher odds to give birth in a public facility [aOR = 6.54 (95% CI: 3.59–11.91)], compared to the poorest women. Rural [aOR = 0.43 (95% CI: 0.28–0.65)] and nomadic [aOR = 0.21 (95% CI: 0.13–0.34)] women had significantly lower odds of giving birth in a public facility than urban women. Sool [aOR = 0.27 (95% CI: 0.15–0.46)] and Sanaag [aOR = 0.16 (95% CI: 0.08–0.30)] showed the lowest odds compared to women from Marodijeh. Higher parity was associated with reduced odds of facility childbirth compared to zero parity, whereas women with formal education had higher odds [aOR = 1.78 (95% CI: 1.23–2.57)] compared to women with no formal education. Decision-making variables and age showed no independent effects.

The full bivariate and multivariable analyses for both outcome variables are shown in Supplementary Table 1 and Supplementary Table 2 provided in the Supplementary material.

#### Measuring inequality

##### Slope-index-of-inequality

**Figure 2:**
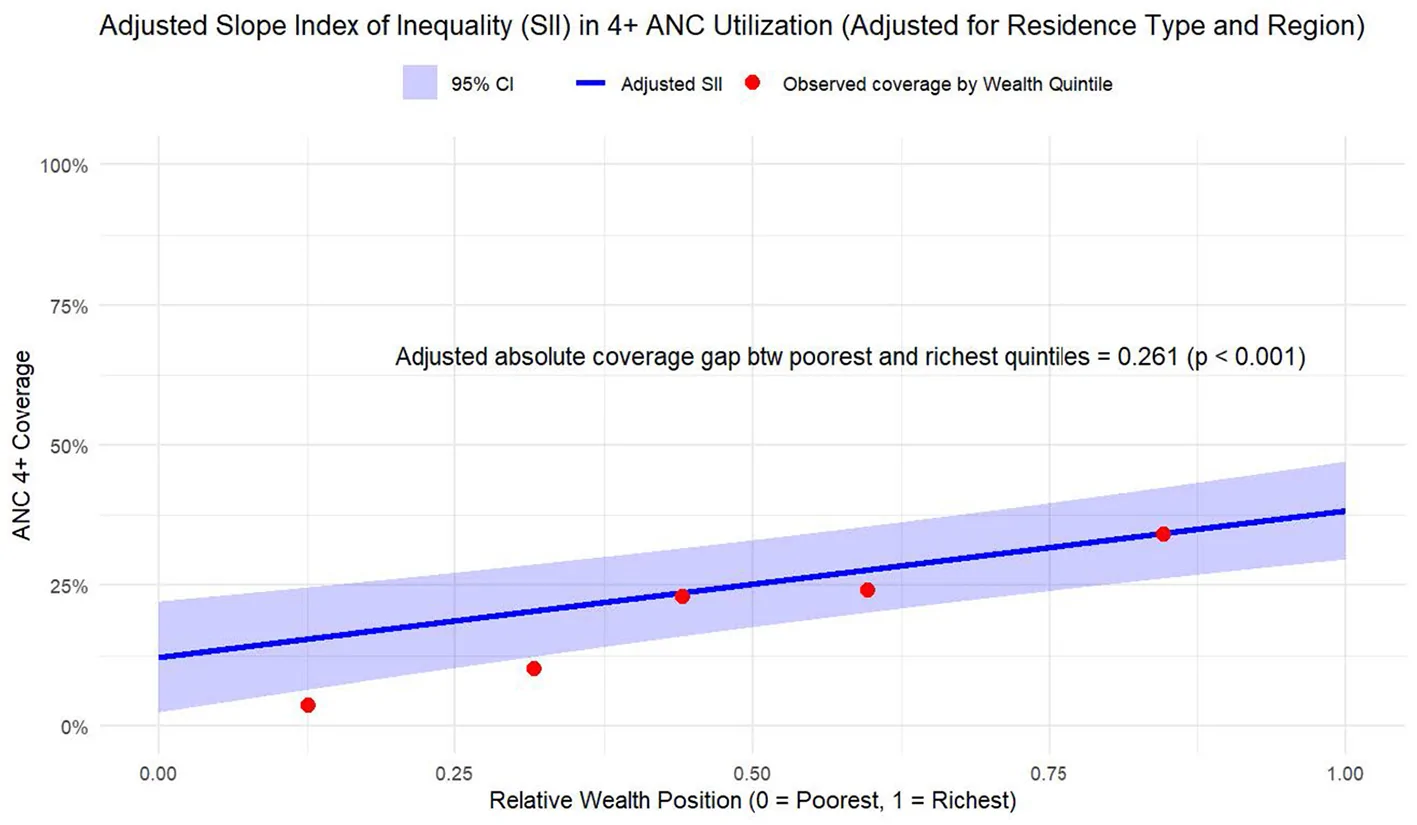
Adjusted SII in 4+ ANC Utilization among ever-married women who had a most recent livebirth in the 5 years preceding the SL-DHS2020.

**Figure 3:**
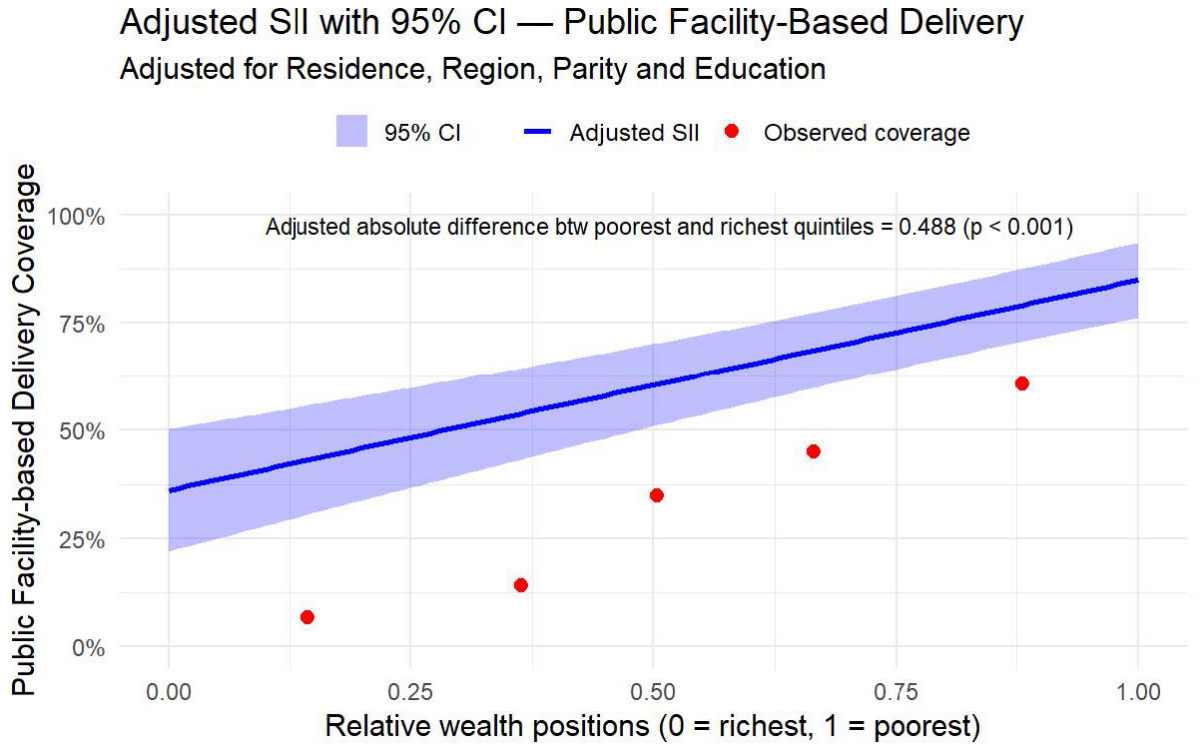
Adjusted SII in public facility-based childbirth coverage among ever-married women who had a most recent livebirth in the 5 years preceding the SL-DHS2020 in Somaliland.

The adjusted SII indicates the absolute difference between the richest quintile and the poorest quintile and adjusts the difference in the middle quintiles. The SII for adequate ANC visits shows a 26-percentage-point difference in coverage between the richest women and the poorest women after adjusting for co-variates (Figure 2). Similarly, public facility-based births show a 49-percentage-point difference between the richest and the poorest women after adjusting for co-variates (Figure 3). All estimates were statistically significant (*p* < 0.001). In practical terms, this means that if the poorest women used adequate ANC and public facility-based birth services at the same rate as the richest women, national coverage of these two indicators could rise by up to 26 and 49 percentage points, respectively, without any change in overall health system capacity.

##### Concentration curves

**Figure 4:**
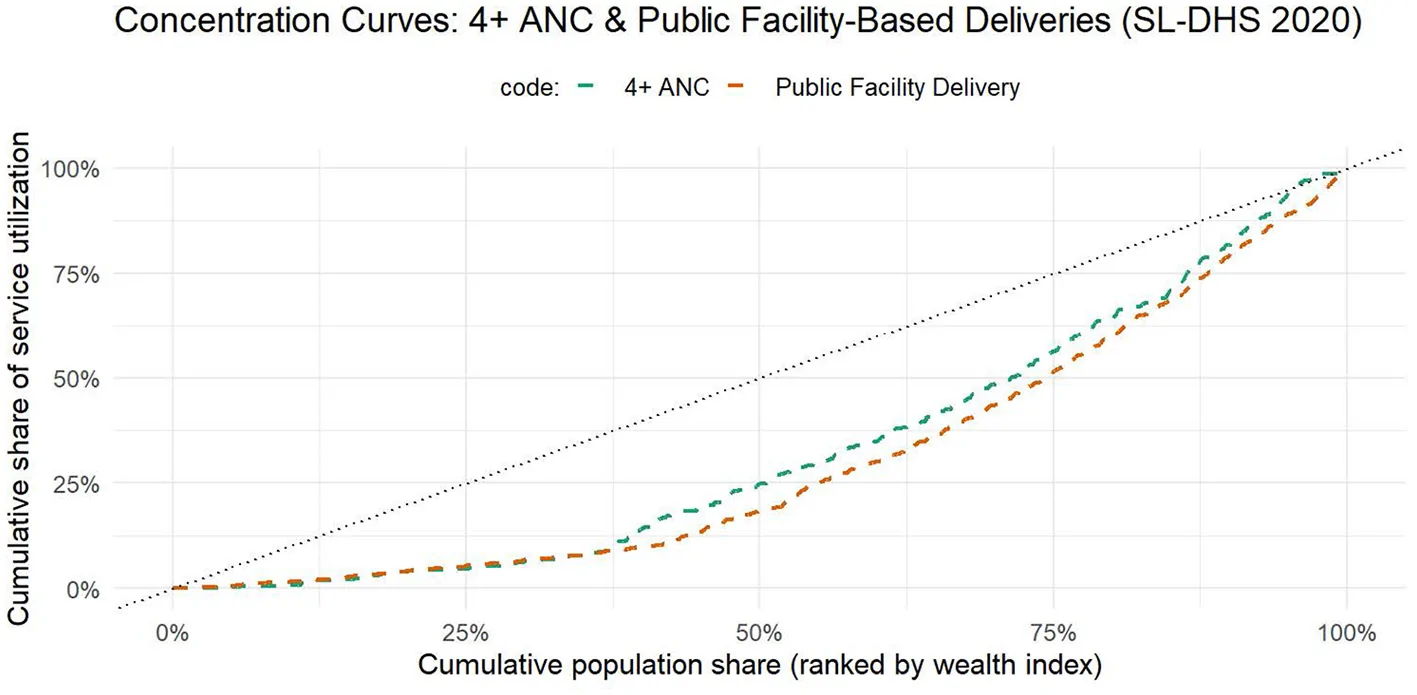
Concentration curves for 4+ ANC and public facility-based births among ever-married women who had a most recent livebirth in the 5 years preceding the SL-DHS2020 in Somaliland.

The concentration curves–as shown in Figure 4–for both adequate ANC visits and public facility-based births lie well below the line of equality, indicating strong pro-rich inequality. Utilization of both services is disproportionately higher among wealthier women, with the poorest groups contributing very little to the cumulative share of service use. The curve for public facility-based births lies slightly farther from the equality line than the ANC curve, suggesting greater inequality in childbirth service use in the public sector than in ANC use (See Figure 4).

##### Concentration indices

The adjusted concentration indices–in Table 4–indicate that both services are used more by wealthier women. For adequate ANC visits, the concentration index of 0.34 (95% CI: 0.33–0.35) shows that use of 4+ ANC is concentrated among the wealthier groups. Similarly, for public facility births, the concentration index was 0.50 (95%CI: 0.49–0.52) and it means a pro-rich pattern of service utilization and concentrated among the wealthier groups. The confidence intervals show statistically significant indices. In practical terms, the concentration index ranges from −1 (service use concentrated entirely among the poorest women) to +1 (concentrated entirely among the richest), with 0 indicating equal use across the wealth distribution; a value of 0.50 for public facility births is very high on this scale and indicates that use is heavily and disproportionately concentrated among wealthier women, while the 0.34 for adequate ANC, though somewhat lower, still reflects substantial pro-rich concentration.

**Table 4:** Concentration indices (Adjusted) of adequate ANC visits and public health facility-based childbirths by the wealth quintiles among ever-married women who had a most recent livebirth in the 5 years preceding the SL-DHS2020 in Somaliland.

Factors	Adjusted concentration indices (95% CI)
Adequate ANC visits (*n* = 3,065)	0.34 (0.33–0.35)
Public facility delivery service (*n* = 2,884)	0.50 (0.49–0.52)

## Discussion

This study assessed the coverage, determinants, and wealth-based inequalities in the utilization of two essential maternal health services in Somaliland: adequate antenatal care (≥4 ANC visits) and public facility-based childbirth, using nationally representative SLDHS 2020 data. The analysis revealed low coverage of both services, with only 20% of women receiving adequate ANC and 32% delivering in public health facilities. Multivariable models indicated wealth as a dominant determinant, with the richest women having nearly four times higher odds of adequate ANC and six times higher odds of public facility childbirths than the poorest, after adjusting for covariates. Finally, significant wealth-based inequality was observed both in absolute terms–with a 26-percentage point difference in adequate ANC visits and a 49-percentage point difference for public facility childbirths between the richest women and the poorest women–and in relative terms–with a concentration index of 0.34 for adequate ANC visits and a concentration index of 0.50 for public facility childbirths. The following paragraphs will interpret these findings and situate them relatively to existing literature.

The coverage of both adequate ANC and public facility-based childbirth observed in this study are lower in Somaliland compared to other countries in SSA (13, 23, 52). A combination of health system, sociocultural and facility-level factors could explain the low coverage of maternal health services utilization in Somaliland. shortage health facilities, especially in non-urban settings, shortages of skilled health workforce (9 qualified core health workers are available for every 10,000 population) and their concentration in urban settings restrict access for large segments of the population (23, 53–58). Additionally, quality of care at the facility level also influence utilization. Qualitative evidence from Somaliland indicates that negative experiences in facilities including disrespectful treatment, lack of privacy, and poor communication, discourage women from seeking institutional delivery, while at the same time, home births assisted by traditional birth attendants may be perceived as more culturally acceptable and supportive (59).

In rural and nomadic areas, geographic distance, weak transportation networks, and effects of recurrent droughts further constrain service availability (23, 40, 42, 54, 60, 61). The mobility of pastoralist communities due to recurrent drought and the constant clan-based conflicts, especially in Eastern regions of Somaliland, disrupt access to continuous maternal care utilization (40, 62, 63). Moreover, decision-making power are often influenced by male household members and female elders, which may delay or discourage preventive care unless complications arise (40, 42). This behavior is more dominant in adolescent mothers who constituted 40% of the sample in this study.

In terms of regions, women in the Eastern regions—Sool and Sanaag—have significantly lower odds of utilizing maternal health services compared to Marodijeh. This could be explained by the chronic insecurity due to clan-based conflicts, recurrent droughts, and limited development in these regions; all of which constrain access to healthcare and education (62–64). Furthermore, multiparity is negatively associated with public facility-based childbirth. This could be explained by the fact that multiparous women often underestimate childbirth risks, face rising logistical challenges including difficulties in leaving household and childcare responsibilities, and experiences of unwelcoming behavior in health facilities which further discourages repeated use of services (65–67).

We also found significant wealth-based inequalities in terms of the utilization of these two services, i.e. wealthier women – regardless of where they reside – are more likely to adequately utilize ANC visits and give birth in a public health facility. This is consistent with the existing evidence from SSA region which shows that maternal health services utilization is wealth-based unequal (2, 12–15, 19, 22, 23, 35, 68, 69). In terms of magnitude, the concentration indices observed in this study (0.34 for adequate ANC and 0.50 for public facility births) place Somaliland among the most unequal settings reported in the region: a 16-country DHS analysis of the maternal continuum of care found concentration indices ranging from 0.04 in South Africa and Liberia to a maximum of 0.34 in Nigeria (13), meaning the level of inequality in public facility-based childbirth documented here exceeds the highest value reported across that 16-country comparison.

The existence of wealth-based inequality in maternal health services in Somaliland is shaped by multiple interconnected variables including affordability of services, distance to health facilities, transportation constraints and costs, and women's autonomy to seek health services (38, 40, 42, 59). Existing local evidence shows that poorer women face significant challenges in accessing services due to long distances, limited and costly transport options, especially women in non-urban settings (23, 39, 40, 55). Additionally, Despite Somaliland's free maternal healthcare policy intended to improve access for poorer populations, unofficial fees for some services – mainly laboratory tests and drugs – continue to exist in most urban districts. Qualitative evidence shows that such charges, and generally user fees, present substantial barriers for poorer women due to unaffordability (40). In addition, mistrust in public maternal health services, driven by perceptions of disrespectful care, lack of privacy, and inconsistent quality, further discourages utilization, particularly among poorer and less educated women whose decisions are influenced by community narratives (40, 59).

Beyond these access-side barriers, the persistence of a strong wealth gradient even after adjusting for covariates suggests that wealth operates as a distinct axis of disadvantage in Somaliland, rather than simply proxying for rural residence or low education. This is consistent with multi-country evidence showing that women's economic status is independently associated with maternal health service use even after accounting separately for their education and empowerment (70), and with concentration-index analyses from Tanzania showing that wealth-related inequality in service utilization does not shrink among more-educated women but is in fact highest among them indicates that education does not substitute for, or neutralize, the effect of household wealth (71). Practically, this implies that interventions targeting geographic access or education alone are unlikely, by themselves, to close the utilization gap. Evidence from across sub-Saharan Africa shows that indirect costs, particularly transport, remain a significant barrier to obstetric care even where services are nominally free and geographically accessible (72), and that the combined direct and indirect costs of antenatal, delivery, and postnatal care can consume a substantial share of household expenditure, a burden poorer households carry disproportionately (73). Equity-oriented maternal health policy in Somaliland therefore needs to explicitly address these direct and indirect costs of care-seeking, alongside continued supply-side investment, if it is to reach the poorest women. In practice, this means pairing supply-side efforts with demand-side financial support for poorer women specifically — such as transport subsidies, cash transfers, or fee waivers extended beyond consultation charges to cover the indirect costs of reaching care — as part of the broader health-financing reforms needed to reduce out-of-pocket payments in the region (74).

This study has several important strengths and some limitations. A key strength is the use of nationally representative DHS data including 3,065 ever-married women, which provides sufficient statistical power. To our knowledge, this is among the first studies in Somaliland to explicitly apply inequality metrics such as absolute differences and concentration curves and indices, to quantify wealth-based inequalities in maternal health service utilization. In addition, the study's explicit focus on public health facility childbirths, differentiated from private facility deliveries, adds contextual relevance by assessing whether services intended to be free and universally accessible are achieving their equity objectives. However, several limitations should be acknowledged. The cross-sectional nature of DHS data limits causal inference. Secondly, reliance on self-reported information which includes often recalling events up to five years prior, has problem in accurately remembering and may introduce a recall bias. Furthermore, the use of the DHS wealth index as a proxy for economic status, while widely accepted, has inherent limitations. As it is based on asset ownership rather than income or expenditure, it may be interpreted differently across urban and rural settings, and assumes equal access to household resources. In patriarchal contexts such as Somaliland, women may have limited control over household assets, weakening the link between measured wealth and service utilization. The index may also overestimate wealth in urban areas and underestimate economic status among rural or nomadic households, where livestock, seasonal income, and mobile assets are poorly captured, potentially leading to misclassification of households' true economic capacity. Additionally, the exclusion of never-married women, necessary because only ever-married women were asked the DHS pregnancy and childbirth questions, may understate the true magnitude of wealth-based inequality, since never-married women are more likely to face additional barriers to maternal healthcare access due to social pressures of becoming mother without any formal marriage. As with any observational analysis, residual confounding from unmeasured factors such as facility readiness, service quality, and individual health-seeking attitudes cannot be ruled out despite adjustment for the measured covariates. Finally, as noted in the Methods, our exclusion of private-facility births—while necessary to isolate access to Somaliland's free public maternal care policy—limits the generalizability of the public facility-based birth outcome to overall institutional delivery coverage, since it does not capture the (plausibly wealthier) births occurring in the private sector; future studies with facility-type-stratified wealth data could quantify this segment directly.

Based on the study's findings, we recommend a focused set of equity-oriented actions to reduce wealth -related disparities in maternal health service utilization in Somaliland. Priority should be given to expanding access in rural and nomadic populations through mobile outreach services linked to functional referral systems, with the explicit objective of reaching poorer women. (23, 40, 42, 59). In parallel, traditional birth attendants (TBAs) should be formally integrated into the maternal health continuum through structured training, supervision, and trust-based referrals that reposition them as advocates and companions for facility-based care (42, 53, 60, 75). Evidence from Somaliland suggests that such engagement can increase skilled birth attendance and strengthen referrals, although sustainability, incentive structures, and ongoing capacity building require careful policy attention (76). In practical terms, this engagement would involve the Ministry of Health Development formally registering TBAs operating in rural and nomadic catchment areas, providing them with short, competency-based training focused on danger-sign recognition and early referral rather than delivery itself, and establishing a structured referral and feedback loop with the nearest public facility, for example through mobile-phone-based communication with health post staff and community health workers, so that TBAs are repositioned as the first link in the referral chain rather than a substitute for facility-based birth.

To address persistent financial barriers, we recommend the explicit linkage of social assistance programs with maternal and child health service. Targeted cash transfers or voucher schemes could help offset transportation costs, opportunity costs, and remaining facility-level expenses for poorer women, particularly in rural and nomadic communities affected by recurrent drought and displacement. These interventions should move beyond short-term shock responsiveness toward transformative social protection that addresses chronic poverty and should be sensitive to humanitarian needs and conflict dynamics (77, 78). Underpinning all recommendations is the need to strengthen health sector governance, including evidence-informed policymaking, improved coordination, and sustainable domestic resource mobilization, particularly as external funding has declined sharply. Future research should prioritize effective coverage and quality of maternal health services, including measures of facility readiness, and should evaluate the role of targeted cash transfers in reducing financial barriers for poorer women in rural and nomadic contexts.

## Conclusion

This study provides among the first nationally representative estimates of wealth-based inequality in maternal health service utilization in Somaliland, using both absolute (Slope Index of Inequality) and relative (concentration index) measures. Coverage of adequate ANC and public facility-based childbirth remains low and is markedly concentrated among wealthier women, with absolute gaps of 26 and 49 percentage points, respectively, between the richest and poorest quintiles. This magnitude of inequality indicates that improving national coverage alone, without deliberate pro-poor strategies targeting rural, nomadic, and conflict-affected populations, is unlikely to translate into equitable gains in maternal health or measurable progress toward universal health coverage.

## Data Availability

Publicly available datasets were analyzed in this study. This data can be found here: https://microdata.nbs.gov.so/index.php/catalog/50/data-dictionary/F3/?offset=5400.

## References

[1] WHO UNICEF UNFPA WBG UNDESA. Trends in Maternal Mortality 2000 to 2023: Estimates by WHO, UNICEF, UNFPA, World Bank Group and UNDESA/Population Division (2025). Available online at: https://www.who.int/publications/i/item/9789240108462 (Accessed April 20, 2025).

[2] Amporfu E Grépin KA. Measuring and explaining changing patterns of inequality in institutional deliveries between urban and rural women in Ghana: a decomposition analysis. Int J Equity Health. (2019) 18:123. doi: 10.1186/s12939-019-1025-z 31399050 PMC6688245

[3] Dickson KS Adde KS Amu H. What influences where they give birth? Determinants of place of delivery among women in rural Ghana. Int J Reprod Med. (2016) 2016:1–8. doi: 10.1155/2016/7203980 PMC5215620 28101522

[4] Moges Mehari A. Asmeret Moges Mehari Levels and Determinants of Use of Institutional Delivery Care Services among Women of Childbearing Age in Ethiopia: Analysis of EDHS 2000 and 2005 Data. Calverton, MD: MEASURE DHS / ICF Macro (2013).

[5] Benova L Macleod D Radovich E Lynch CA Campbell OMR. Should I stay or should I go? : Consistency and switching of delivery locations among new mothers in 39 Sub-Saharan African and South/Southeast Asian countries. Health Policy Plan. (2017) 32:1294–308. doi: 10.1093/heapol/czx087 28981668 PMC5886240

[6] Rahman MA Sultana S Kundu S Islam MA Roshid HO Khan ZI . Trends and patterns of inequalities in using facility delivery among reproductive-age women in Bangladesh: a decomposition analysis of 2007-2017 Demographic and Health Survey data. BMJ Open. (2022) 12:e065674. doi: 10.1136/bmjopen-2022-065674 PMC9806084 36581408

[7] Graham W Bell JS Bullough CHW. Can Skilled Attendance at Delivery Reduce Maternal Mortality in Developping Countries?. Available online at: https://www.researchgate.net/publication/239924607_Can_Skilled_Attendance_at_Delivery_Reduce_Maternal_Mortality_in_Developping_Countries (Accessed July 18, 2026).

[8] Miltenburg AS Roggeveen Y Shields L Van Elteren M Roosmalen J Van Stekelenburg J . Impact of birth preparedness and complication readiness interventions on birth with a skilled attendant: a systematic review. PLoS ONE. (2015) 10:e0143382. doi: 10.1371/journal.pone.0143382 26599677 PMC4658103

[9] UNICEF. Antenatal care - UNICEF DATA (2025). Available online at: https://data.unicef.org/topic/maternal-health/antenatal-care/ (Accessed February 20, 2025).

[10] UNICEF. Delivery Care - UNICEF DATA (2024). Available online at: https://data.unicef.org/topic/maternal-health/delivery-care/(Accessed June 13, 2025).

[11] Wealth Health Organization. State of Inequality: Reproductive, Maternal, Newborn and Child Health (2015). Available online at: https://www.who.int/docs/default-source/gho-documents/health-equity/state-of-inequality/state-of-inequality-reproductive-maternal-new-born-and-child-health.pdf (Accessed August 03, 2025).

[12] Abekah-Nkrumah G. Trends in utilisation and inequality in the use of reproductive health services in Sub-Saharan Africa. BMC Public Health. (2019) 19:1541. doi: 10.1186/s12889-019-7865-z 31752773 PMC6873654

[13] Asefa A Gebremedhin S Marthias T Nababan H Christou A Semaan A . Wealth-based inequality in the continuum of maternal health service utilisation in 16 sub-Saharan African countries. Int J Equity Health. (2023) 22:203. doi: 10.1186/s12939-023-02015-0 37784140 PMC10544383

[14] Alam N Hajizadeh M Dumont A Fournier P. Inequalities in maternal health care utilization in sub-saharan African countries: a multiyear and multi-country analysis. PLoS ONE. (2015) 10:e0120922. doi: 10.1371/journal.pone.0120922 25853423 PMC4390337

[15] Obse AG Ataguba JE. Explaining socioeconomic disparities and gaps in the use of antenatal care services in 36 countries in sub-Saharan Africa. Health Policy Plan. (2021) 36:651–61. doi: 10.1093/heapol/czab036 33751100 PMC8173665

[16] Tey NP Lai SL. Correlates of and barriers to the utilization of health services for delivery in South Asia and Sub-Saharan Africa. Sci World J. (2013) 2013:423403. doi: 10.1155/2013/423403 PMC3830882 24288482

[17] Samuel O Zewotir T North D. Decomposing the urban–rural inequalities in the utilisation of maternal health care services: evidence from 27 selected countries in Sub-Saharan Africa. Reprod Health. (2021) 18:216. doi: 10.1186/s12978-021-01268-8 34717668 PMC8557532

[18] Bobo FT Yesuf EA Woldie M. Inequities in utilization of reproductive and maternal health services in Ethiopia. Int J Equity Health. (2017) 16:105. doi: 10.1186/s12939-017-0602-2 28629358 PMC5477250

[19] Goli S Nawal D Rammohan A Sekher T V Singh D. Decomposing the socioeconomic inequality in utilization of maternal health care services in selected countries of south Asia and sub-Saharan Africa. J Biosoc Sci. (2018) 50:725–48. doi: 10.1017/S0021932017000530 29081310

[20] Makate M Makate C. The evolution of socioeconomic status-related inequalities in maternal health care utilization: evidence from Zimbabwe, 1994–2011. Glob Health Res Policy. (2017) 2:1. doi: 10.1186/s41256-016-0021-8 29202069 PMC5683384

[21] Ambel AA Andrews C Bakilana AM Foster EM Khan Q Wang H. Examining changes in maternal and child health inequalities in Ethiopia. Int J Equity Health. (2017) 16:152. doi: 10.1186/s12939-017-0648-1 28830454 PMC5568328

[22] Sanoussi Y. Measurement and analysis of inequality of opportunity in access of maternal and child health care in Togo. BMC Health Serv Res. (2017) 17:699. doi: 10.1186/s12913-017-2647-8 29219086 PMC5773902

[23] Morrison J Malik SMMR. Health equity in Somalia? An evaluation of the progress made from 2006 to 2019 in reducing inequities in maternal and newborn health. Int J Equity Health. (2024) 23:46. doi: 10.1186/s12939-023-02092-1 38443921 PMC10916226

[24] Zere E Oluwole D Kirigia JM Mwikisa CN Mbeeli T. Inequities in skilled attendance at birth in Namibia: a decomposition analysis. BMC Pregnancy Childbirth. (2011) 11:34. doi: 10.1186/1471-2393-11-34 21569585 PMC3118957

[25] Woldemicael G. Do women with higher autonomy seek more maternal and child health-care? Evidence from Ethiopia and Eritrea. Health Care Women Int. (2007) 37:599–620. doi: 10.4054/MPIDR-WP-2007-035 20526926

[26] Say L Raine R. A systematic review of inequalities in the use of maternal health care in developing countries: examining the scale of the problem and the importance of context. Bull World Health Organ. (2007) 85:812–9. doi: 10.2471/BLT.06.035659 18038064 PMC2636485

[27] Doctor HV Nkhana-Salimu S Abdulsalam-Anibilowo M. Health facility delivery in sub-Saharan Africa: successes, challenges, and implications for the 2030 development agenda. BMC Public Health. (2018) 18:765. doi: 10.1186/s12889-018-5695-z 29921275 PMC6011205

[28] Kruk ME Rockers PC Mbaruku G Paczkowski MM Galea S. Community and Health System Factors Associated with Facility Delivery in Rural Tanzania: a Multilevel Analysis (2010). Available online at: https://openurl.ebsco.com/contentitem/cat01877a:itm.68018?sid=ebsco:plink:crawler&id=ebsco:cat01877a:itm.68018&crl=c (Accessed February 21, 2025). 10.1016/j.healthpol.2010.05.002 20537423

[29] Abel Ntambue ML Françoise Malonga K Dramaix-Wilmet M Donnen P. Determinants of maternal health services utilization in urban settings of the democratic republic of Congo - a case study of Lubumbashi city. BMC Pregnancy Childbirth. (2012) 12:66. doi: 10.1186/1471-2393-12-66 22780957 PMC3449182

[30] Muhajarine N Shakurun N Ahmed MS Andre F Chicumbe S. Inequalities and factors associated with maternal healthcare services utilisation in Mozambique: evidence from the demographic and health survey 2022–2023. BMJ Glob Health. (2025) 10:e018121. doi: 10.1136/bmjgh-2024-018121 40412813 PMC12104887

[31] Boerma T Eozenou P Evans D Evans T Kieny MP Wagstaff A. Monitoring progress towards universal health coverage at country and global levels. PLoS Med. (2014) 11:e1001731. doi: 10.1371/journal.pmed.1001731 25243899 PMC4171369

[32] WHO. Monitoring Health-Related SDGs. (2017). https://www.who.int/publications/i/item/background-paper-for-the-regional-technical-consultation-on-monitoring-the-health-related-sustainable-development-goals-(sdgs)

[33] Wealth Health Organization. Handbook on Health Inequality Monitoring: with a Special Focus on Low- and Middle-Income Countries (2013). Available online at: https://www.who.int/publications/i/item/9789241548632 (Accessed March 18, 2025).

[34] Leventhal DGP Crochemore-Silva I Vidaletti LP Armenta-Paulino N Barros AJD Victora CG. Delivery channels and socioeconomic inequalities in coverage of reproductive, maternal, newborn, and child health interventions: analysis of 36 cross-sectional surveys in low-income and middle-income countries. Lancet Glob Health. (2021) 9:e1101–9. doi: 10.1016/S2214-109X(21)00204-7 34051180 PMC8295042

[35] Wehrmeister FC Fayé CM da Silva ICM Amouzou A Ferreira LZ Jiwani SS . Wealth-related inequalities in the coverage of reproductive, maternal, newborn and child health interventions in 36 countries in the African region. Bull World Health Organ. (2020) 98:394. doi: 10.2471/BLT.19.249078 32514213 PMC7265922

[36] Org SU. Transforming Our World: The 2030 Agenda For Sustainable Development United Nations United Nations Transforming Our World: The 2030 Agenda For Sustainable Development (2015). Available online at: https://digitallibrary.un.org/record/3923923?ln=en&v=pdf (Accessed June 17, 2025).

[37] Arcaya MC Arcaya AL Subramanian S V. Inequalities in health: definitions, concepts, and theories. Glob Health Action. (2015) 8:10. doi: 10.3402/gha.v8.27106 26112142 PMC4481045

[38] Ministry of Planning and National Development. The Somaliland Health and Demographic Survey 2020 (2020). Available online at: www.somalilandmohd.com (Accessed February 09, 2025).

[39] Abdiwali SA Adesina OA Fekadu GA. Antenatal care services uptake and associated factors in Somaliland: further analysis of the 2020 Somaliland demographic health survey. Open Public Health J. (2024) 17:. doi: 10.2174/0118749445285088240227053051

[40] Abdiwali SA Adesina OA Fekadu GA Geta TG. Barriers and facilitators to antenatal care services utilisation in Somaliland: a qualitative study. BMJ Open. (2024) 14:e085073. doi: 10.1136/bmjopen-2024-085073 39488416 PMC11535687

[41] Ministry of Health Development S. Somaliland Health Sector Strategic Plan II 2017–2021 (2017). Available online at: https://moh.gov.so/so/wp-content/uploads/2023/06/Health-Sector-Strategic-Plan-II-2017-%E2%80%93-2021.-SLSS.pdf (Accessed February 09, 2025).

[42] Abdi KA Jayamohan MK Adem M. The nexus between poverty and maternal healthcare utilization with a focus on antenatal care visits and choice of place of birth in Somaliland. Front Public Health. (2024) 12:1417883. doi: 10.3389/fpubh.2024.1417883 39377007 PMC11457695

[43] Mohamed M Abdeeq BA Mohamed AI Jama HA Tafese F Getachew M. Level of institutional delivery service utilization and associated factors among women who gave birth in the past 12 months, Ga'an libah district, Marodijeh region, Somaliland: a community-based cross-sectional study. BMC Health Serv Res. (2024) 24:1085. doi: 10.1186/s12913-024-11330-3 39289673 PMC11409708

[44] Alkhawaldeh A Albashtawy M Rayan A Abdalrahim A Musa A Eshah N . Application and use of Andersen's behavioral model as theoretical framework: a systematic literature review from 2012–2021. Iran J Public Health. (2023) 52:1346. doi: 10.18502/ijph.v52i7.13236 37593505 PMC10430393

[45] Alyafei A Easton-Carr R. The Health Belief Model of Behavior Change. Treasure Island, FL: StatPearls (2024). 39163427

[46] Beauchamp MR Crawford KL Jackson B. Social cognitive theory and physical activity: mechanisms of behavior change, critique, and legacy. Psychol Sport Exerc. (2019) 42:110–7. doi: 10.1016/j.psychsport.2018.11.009

[47] Schunk DH Pajares F. Self-Efficacy Beliefs. Int Encyclopedia Educ. (2009) 2010:668–72. doi: 10.1016/B978-0-08-044894-7.00620-5

[48] Lewis TP Ndiaye Y Manzi F Kruk ME. Associations between women's empowerment, care seeking, and quality of malaria care for children: a cross-sectional analysis of demographic and health surveys in 16 sub-Saharan African countries. J Glob Health. (2022) 12:04025. doi: 10.7189/jogh.12.04025 35356662 PMC8932460

[49] Barros AJD Victora CG. Measuring coverage in MNCH: determining and interpreting inequalities in coverage of maternal, newborn, and child health interventions. PLoS Med. (2013). doi: 10.1371/journal.pmed.1001390 23667332 PMC3646214

[50] O'Donnell O van Doorslaer E Wagstaff A Lindelow M. Analyzing Health Equity Using Household Survey Data. Washington, DC: Analyzing Health Equity Using Household Survey Data. (2007).

[51] Erreygers G Van Ourti T. Measuring socioeconomic inequality in health, health care and health financing by means of rank-dependent indices: a recipe for good practice. J Health Econ. (2011) 30:685–94. doi: 10.1016/j.jhealeco.2011.04.004 21683462 PMC3158909

[52] Yaya S Ghose B. Global inequality in maternal health care service utilization: implications for sustainable development goals. Health Equity. (2019) 3:145–54. doi: 10.1089/heq.2018.0082 31289773 PMC6608688

[53] Abdikarim H Muse AH Hassan MA Muse YH. Prevalence and determinants of home delivery among pregnant women in Somaliland: Insights from SLDHS 2020 data. Aten Primaria. (2025) 57:103082. doi: 10.1016/j.aprim.2024.103082 39288728 PMC11420480

[54] Mohamed AA Akin A Mihciokur S Üner S Gele A. Level of completion of maternity continuum of care among ever-married women: an analysis of Somalia's health and demographic survey 2020. PLoS Global Public Health. (2025) 5:e0004102. doi: 10.1371/journal.pgph.0004102 39792806 PMC11723541

[55] Geta TG Abdiwali SA Farah MM Assefa DZ Arusi TT. Multilevel analysis on prevalence and associated factors of modern contraceptive uptake in Somaliland: based on the Somaliland health and demographic survey 2020. Reprod Health . (2024) 21:67. doi: 10.1186/s12978-024-01786-1 38773601 PMC11110236

[56] Ministry of Planning and National Development 2023. National Development Plan III 2023–2027 (2023). Available online at: https://www.informea.org/en/content/legislation/somaliland-national-development-plan-iii-2023-2027 (Accessed February 09, 2025).

[57] Ministry of Health Development 2023. Health Sector Strategic Plan 2023–2027 (2023). Available online at: https://www.health.go.ke/sites/default/files/2024-10/DRAFT%20STRATEGIC%20PLAN%202023-2027%20SDPH%26PS%20Sept%2027-4.pdf (Accessed February 09, 2025).

[58] Ministry of Health Development 2023. Somaliland Comprehensive Health and Nutrition Report (2023). Available online at: www.somalilandmohd.com (Accessed February 09, 2025).

[59] Egal JA Essa A Yusuf R Osman F Ereg D Klingberg-Allvin M . A lack of reproductive agency in facility-based births makes home births a first choice regardless of potential risks and medical needs—a qualitative study among multiparous women in Somaliland. Glob Health Action. (2022) 15:2054110. doi: 10.1080/16549716.2022.2054110 35389334 PMC9004503

[60] Sheikh NS Hussein AM Mohamed SS Gele A. Does living in major towns favor institutional delivery in Somalia? Front Glob Womens Health. (2024) 5:1216290. doi: 10.3389/fgwh.2024.1216290 39119357 PMC11306125

[61] Miikkulainen A Abdirahman Mohamud I Aqazouz M Abdullahi Suleiman B Sheikh Mohamud O Ahmed Mohamed A . Antenatal care utilization and its associated factors in Somalia: a cross-sectional study. BMC Pregnancy Childbirth. (2023) 23:581. doi: 10.1186/s12884-023-05871-4 37573367 PMC10422779

[62] Rift Valley Institute Research Project Funded by the LOGiCA trust fund of the World Bank A. The Impact of War on Somali Men - An Inception Study (2015). Available online at: www.logica-wb.net (Accessed June 02, 2025).

[63] ISS- East Africa Report - Vol. 2019 No 27. Overlapping claims by Somaliland and Puntland : the case of Sool and Sanaag | ISS East Africa Report (2019). Available online at: https://journals.co.za/doi/abs/10.10520/EJC-1fda5c2280 (Accessed June 05, 2025).

[64] Hoehne MV. Between Somaliland and Puntland Marginalization, Militarization and Conflicting Political Visions Rift Valley Institute | Contested Borderlands (2015). Available online at: www.riftvalley.net (Accessed June 02, 2025).

[65] Moyer CA Mustafa A. Drivers and deterrents of facility delivery in sub-Saharan Africa: a systematic review. Reprod Health. (2013) 10:40. doi: 10.1186/1742-4755-10-40 23962135 PMC3751820

[66] Dunlop CL Benova L Campbell O. Effect of maternal age on facility-based delivery: analysis of first-order births in 34 countries of sub-Saharan Africa using demographic and health survey data. BMJ Open. (2018) 8:e020231. doi: 10.1136/bmjopen-2017-020231 29654024 PMC5905782

[67] Kyei-Nimakoh M Carolan-Olah M McCann TV. Access barriers to obstetric care at health facilities in sub-Saharan Africa-a systematic review. Syst Rev. (2017) 6:110. doi: 10.1186/s13643-017-0503-x 28587676 PMC5461715

[68] Bintabara D Mwampagatwa I. Socioeconomic inequalities in maternal healthcare utilization: an analysis of the interaction between wealth status and education, a population-based surveys in Tanzania. PLoS Global Public Health. (2023) 3:e0002006. doi: 10.1371/journal.pgph.0002006 37310944 PMC10263315

[69] Selebano KM Ataguba JE. Decomposing socio-economic inequalities in antenatal care utilisation in 12 Southern African development community countries. SSM Popul Health. (2022) 1:17. doi: 10.1016/j.ssmph.2021.101004 34988282 PMC8703074

[70] Ahmed S Creanga AA Gillespie DG Tsui AO. Economic status, education and empowerment: implications for maternal health service utilization in developing countries. PLoS ONE. (2010) 5:e11190. doi: 10.1371/journal.pone.0011190 20585646 PMC2890410

[71] Bintabara D Mwampagatwa I. Socioeconomic inequalities in maternal healthcare utilization: an analysis of the interaction between wealth status and education, a population-based surveys in Tanzania. PLoS Glob Public Health. (2023) 3:e0002006. doi: 10.1371/journal.pgph.0002006 37310944 PMC10263315

[72] Kyei-Nimakoh M Carolan-Olah M McCann TV. Access barriers to obstetric care at health facilities in sub-Saharan Africa-a systematic review. Syst Rev. (2017) 6:110. doi: 10.1186/s13643-017-0503-x 28587676 PMC5461715

[73] Banke-Thomas A Ayomoh FI Abejirinde IOO Banke-Thomas O Eboreime EA Ameh CA. Cost of utilising maternal health services in low- and middle-income countries: a systematic review. Int J Health Policy Manag. (2021) 10:564–77. doi: 10.34172/ijhpm.2020.104 32610819 PMC9278371

[74] Farih OA Mohamed SH Abdillahi AM Abokor AH Ali MA Muse AH . Financial barriers and inequalities in healthcare access across East Africa: evidence from demographic and health surveys. Front Reprod Health. (2026) 7:1730560. doi: 10.3389/frph.2025.1730560 41623701 PMC12852407

[75] Mothupi M Ahmed MA Mohamud AM Dalmar A Jimale MAO Abdullahi H . Maternal and newborn health prioritization in post-transition Somalia: analysis of key stakeholder perspectives at the federal level. SSM Health Syst. (2025) 4:100072. doi: 10.1016/j.ssmhs.2025.100072

[76] Pyone T Adaji S Madaj B Woldetsadik T Van Den Broek N. Changing the role of the traditional birth attendant in Somaliland. Int J Gynecol Obstet. (2014) 127:41–6. doi: 10.1016/j.ijgo.2014.04.009 24938771

[77] Birch I. Conflict-Sensitive Social Protection: Somalia Country Report. Brighton: Institute of Development Studies (2023).

[78] Lowe C Hagen-Zanker J. Linking social protection and humanitarian systems to respond to forced displacement. IDS Bull. (2024) 55:135–50. doi: 10.19088/1968-2024.126

