# Supplementary material for: Wealth-based inequality in maternal health service utilization among ever-married women of reproductive age in Somaliland; secondary analysis of the 2020 Demographic and Health Survey
Source: Front Public Health. 2026 Aug 19;14:1899022. doi: 10.3389/fpubh.2026.1899022 (PMC13533987; doi:10.3389/fpubh.2026.1899022)
Supplement: Supplementary file 1 [file Table_1.docx]

Additional Table 1 - Results of the bivariate and multivariable regression analysis of adequate ANC visits

| **Adequate ANC Visits (4 and more ANC Visits) (n = 3,065)** | | | | | |
| --- | --- | --- | --- | --- | --- |
| **Sociodemographic Determinants** | **Bivariate Results** | | **Multivariate Analysis Results** | | |
|  | **cOR (95% CI)** | **P-Value** | **aOR (95% CI)**  **Regression Model 1** | | **aOR**  **Regression Model 2** |
| **Wealth Index** | | | | |  |
| Poorest | Ref | < 0.001 | Ref | | Ref |
| Poorer | 3.11 (1.57 – 6.17) |  | 1.40 (0.70 – 2.80) | | 1.57 (0.81 – 3.05) |
| Middle | 8.15 (4.27 – 15.55) |  | 2.07 (1.03 – 4.17) | | 2.60 (1.34 – 3.05) |
| Richer | 8.72 (4.91 – 15.48) |  | 2.22 (1.11 – 4.44) | | 2.82 (1.49 – 5.31) |
| Richest | 14.14 (8.14 – 24.58) |  | 3.08 (1.54 – 6.16) | | 3.98 (2.20 – 7.17) |
| **Residence type** | | | | | |
| Urban | Ref | < 0.001 | Ref | | Ref |
| Rural | 0.53 (0.31 – 0.54) |  | 0.92 (0.61 – 1.41) | | 0.87 (0.57 – 1.33) |
| Nomadic | 0.02 (0.01 – 0.05) |  | 0.10 (0.04 – 0.22) | | 0.10 (0.04 – 0.21) |
| **Region of Residence** | | | | | |
| Marodijeh | Ref | <0.001 | Ref | | Ref |
| Awdal | 1.10 (0.68 – 1.78) |  | 1.84 (1.17 – 2.89) | | 1.81 (1.14 – 2.88) |
| Sahil | 0.62 (0.38 – 1.03) |  | 1.08 (0.71 – 1.66) | | 0.93 (0.58 – 1.47) |
| Togdheer | 0.95 (0.53 – 1.72) |  | 1.34 (0.83 – 2.16) | | 1.18 (0.72 – 1. 93) |
| Sool | 0.18 (0.12 – 0.31) |  | 0.44 (0.25 – 0.79) | | 0.42 (0.23 – 0.75) |
| Sanaag | 0.21 (0.13 – 0.36) |  | 0.39 (0.24 – 0.63) | | 0.40 (0.24 – 0.67) |
| **Education of the mother** | | | | |  |
| No formal Education | Ref | < 0.001 | Ref | |  |
| At Least has a Primary Education | 2.54 (1.79 – 3.61) |  | 1.36 (0.92 – 2.00) | |  |
| **Husband’s Education** | | | | |  |
| No formal education | Ref | <0.001 | Ref | |  |
| At Least has a Primary Education | 2.57 (2.00 – 3.30) |  | 1.02 (0.76 – 1.37) | |  |
| **Parity prior to the last pregnancy** | | | | |  |
| 0 parity | Ref | 0.08 | Ref | |  |
| 1 – 3 parities | 0.96 (0.59 – 1.57) |  | 1.34 (0.78 – 2.31) | |  |
| 4+ parities | 0.69 (0.46 – 1.05) |  | 0.96 (0.56 – 1.64) | |  |
| **Decision of seeking care in general** | | | | |  |
| Independently by the mother or in consultation with her husband | Ref | 0.001 | Ref | |  |
| Husband and others ONLY | 0.60 (0.43 – 0.83) |  | 0.81 (0.58 – 1.13) | |  |
| **Age of the mother at the time of the most recent livebirth (in groups)** | | | |  |  |
| 14 – 19 | Ref | 0.72 |  |  |  |
| 20 – 29 | 1.13 (0.78 – 1.63) |  |  |  |  |
| 30 and above | 0.89 (0.34 – 2.28) |  |  |  |  |

Additional Table 2 - Results of Bivariate and Multivariable Regression Analysis of the Public Facility-based Births

| **Public Health Facility-based Childbirths (n = 2,884)** | | | | | |
| --- | --- | --- | --- | --- | --- |
| **Sociodemographic Determinants** | **Bivariate Results** | | **Multivariate Logistic Regression Results** | | |
|  | **cOR (95% CI)** | **P Value** | **aOR (95% CI)**  **Regression Model 1** | **aOR (95% CI)**  **Regression Model 2** | **aOR (95% CI)**  **Regression Model 3** |
| **Wealth Index** | | | | | |
| Poorest | Ref | < 0.001 | Ref | Ref | Ref |
| Poorer | 2.30 (1.47 – 3.60) |  | 1.65 (0.89 – 3.07) | 1.67 (0.89 – 3.11) | 1.79 (0.99 – 3.25) |
| Middle | 7.54 (5.02 – 11.30) |  | 2.88 (1.61 – 5.14) | 1.90 (1.62 – 5.19) | 3.32 (1.94 – 5.66) |
| Richer | 11.52 (7.52 – 17.66) |  | 4.18 (1.92 – 9.14) | 4.08 (1.88 – 8.86) | 4.87 (2.47 – 9.55) |
| Richest | 21.68 (14.51 – 32.40) |  | 3.89 (2.56 – 9.31) | 4.98 (2.62 – 9.48) | 6.54 (3.59 – 11.91) |
| **Residence type** | | | | | |
| Urban | Ref | <0.001 | Ref | Ref | Ref |
| Rural | 0.30 (0.18 – 0.51) |  | 0.41 (0.28 – 0.59) | 0.40 (0.28 – 0.58) | 0.43 (0.28 – 0.65) |
| Nomadic | 0.04 (0.03 – 0.06) |  | 0.20 (0.12 – 0.34) | 0.19 (0.11 – 0.32) | 0.21 (0.13 – 0.34) |
| **Region of Residence** | | | | | |
| Marodijeh | Ref | <0.001 | Ref | Ref | Ref |
| Awdal | 0.84 (0.39 – 1.79) |  | 1.39 (0.83 – 2.34) | 1.33 (0.80 – 2.22) | 1.42 (0.79 – 2.56) |
| Sahil | 0.78 (0.39 – 1.56) |  | 1.24 (0.70 – 2.20) | 1.22 (0.69 – 2.17) | 1.17 (0.66 – 2.08) |
| Togdher | 0.39 (0.19 – 0.82) |  | 0.37 (0.21 – 0.64) | 0.35 (0.20 – 0.61) | 0.32 (0.18 – 0.56) |
| Sool | 0.15 (0.09 – 0.30) |  | 0.30 (0.18 – 0.52) | 0.28 (0.16 – 0.49) | 0.27 (0.15 – 0.46) |
| Sanaag | 0.13 (0.06 – 0.27) |  | 0.13 (0.07 – 0.25) | 0.13 (0.07 – 0.25) | 0.16 (0.08 – 0.30) |
| **Parity prior to the last pregnancy** | | | | |  |
| 0 parity | Ref | 0.001 | Ref | Ref | Ref |
| 1 – 3 parities | 0.66 (0.44 – 0.98) |  | 0.56 (0.34 – 0.91) | 0.58 (0.36 – 0.94) | 0.65 (0.42 – 1.00) |
| 4+ parities | 0.48 (0.31 – 0.73) |  | 0.39 (0.22 – 0.69) | 0.40 (0.24 – 0.68) | 0.48 (0.29 – 0.76) |
| **Education Level of the mother** | | | | |  |
| No formal education | Ref | <0.001 | Ref | Ref | Ref |
| At Least has a Primary Education | 3.59 (2.72 – 4.76) |  | 1.78 (1.20 – 2.63) | 1.78 (1.22 – 2.59) | 1.78 (1.23 – 2.57) |
| **Husband’s Education** | | | | |  |
| No formal education | Ref | <0.001 | Ref | Ref |  |
| At Least has a Primary Education | 4.04 (2.93 – 5.58) |  | 1.46 (0.94 – 2.25) | 1.48 (0.96 – 2.27) |  |
| **Age of the mother at the time of the most recent live birth (in groups)** | | | |  |  |
| 14 – 19 | Ref | 0.009 | Ref |  |  |
| 20 – 29 | 1.53 (1.18 – 2.00) |  | 1.25 (0.94 – 1.66) |  |  |
| 30 and above | 1.36 (0.69 – 2.67) |  | 0.98 (0.43 – 2.24) |  |  |
| **Decision of seeking care in general** | | | |  |  |
| Independently by the mother or in consultation with her husband | Ref | <0.001 | Ref |  |  |
| Husband and others ONLY | 0.53 (0.41 – 0.69) |  | 0.72 (0.51 – 1.01) |  |  |
